# Supplementary material for: Content-rich biological network constructed by mining PubMed abstracts
Source: BMC Bioinformatics. 2004 Oct 8;5:147. doi: 10.1186/1471-2105-5-147 (PMC528731; doi:10.1186/1471-2105-5-147)
Supplement: Additional File 2 — The original results of the above study (non-essential files are deleted to keep the file size under the limit set by BMC bioinformatics). [file 1471-2105-5-147-S2.bz2 › chilibotAdditionalFile2/dip05/538ID11085509E10890/html/AR_BRCA1.html]

 


 **AR** and **BRCA1** 
  
Found 28 abstracts in PubMed, retrieved 05.  
 

 What does Google say? 
 PDF only 
| .edu only 

---

**Interactive relationship** (e.g. stimulation, inhibition, etc)

**Neutral relationship**- RESULTS The  **AR**  was only present in 12% of  **BRCA1**  mutated tumors, with mutations located at the C terminal half of the  **BRCA1**  gene.  Ref: 12779088 Breast Cancer Res Treat, 2003
- The loss of the  **AR**  expression, as shown by immunohistochemistry, together with the observed loss of other steroid hormone receptors in  **BRCA1**  mutated tumors may lead to a hormone independent growth or to anti hormone resistant growth of these tumors.  Ref: 12779088 Breast Cancer Res Treat, 2003

**Non-interactive relationship** (e.g. studied together, co-existance, homology, etc.)

- Since this growth factor is induced by androgens, we studied the androgen receptor  [ **AR** ]   **AR**  expression in BRCA  [ **BRCA1** ]  mutated tumors and in matched sporadic breast tumors.  Ref: 12779088 Breast Cancer Res Treat, 2003
- METHODS Paraffin embedded breast tumors of carriers of a  **BRCA1**  mutation n=41, median age of patients at time of surgery was 41 years range 28 59 years or a BRCA2 mutation n=14, median age 41 years range 31 85 years were analyzed for the presence of ER alpha, PR, P53 and  **AR**  using standard immunohistochemical techniques.  Ref: 12779088 Breast Cancer Res Treat, 2003
- BRCA2 not only associates with  **AR**  and GRIP1 but also cooperates with both the histone acetyltransferase P CAF and  **BRCA1**  to enhance  **AR**  and GRIP1 mediated transactivation.  Ref: 12756300 Proc Natl Acad Sci U S A< MedlineTA>Proc Natl Acad Sci U S A, 2003
